# Influence of surface pretreatment using cold-active atmospheric pressure plasma on bond strength of CAD-CAM-fabricated hybrid ceramic crowns: an in-vitro study

**DOI:** 10.1186/s40729-024-00584-5

**Published:** 2024-12-23

**Authors:** Georgi Kostadinov, Carolin-Isabel Görgen, Irene Schmidtmann, Gernot Weibrich, Samir Abou-Ayash, Stefan Wentaschek

**Affiliations:** 1 Department for Prosthetic Dentistry and Materials, University Medical Centre, Augustusplatz 2, 55131 Mainz, Germany; 2 Institute for Medical Biostatistics, Epidemiology and Informatics, University Medical Centre, Obere Zahlbacher Str. 69, 55131 Mainz, Germany; 3 Department of Reconstructive Dentistry and Gerodontology, School of Dental Medicine, University of Bern, Freiburgstrasse 7, 3007 Bern, Switzerland

**Keywords:** Plasma, Hydrofluoric acid, Hybrid ceramic, Silane, Pull-off bond strength

## Abstract

**Purpose:**

This study assesses the impact of Cold Atmospheric Pressure Plasma (CAP) pretreatment on the bond strength of two-piece hybrid ceramic abutment crowns in implant dentistry. The objective is to ascertain whether CAP can be employed as an alternative or complementary technique to conventional methods.

**Methods:**

80 titanium bases and 80 VITA ENAMIC^®^ polymer-infiltrated ceramic network (PICN) crowns were divided into 8 groups (n = 10) based on different surface pretreatments of the crowns before cementation: no treatment (A), hydrofluoric acid (HF) (B), HF and silane (C), silane (D), CAP (AP), HF and CAP (BP), HF, CAP, and silane (CP), and CAP and silane (DP). Bond strength (BS) was measured after thermocycling (5000 cycles at 5 °C/55 °C), and statistical analysis was performed using three-way ANOVA.

**Results:**

The highest bond strength (BS) was recorded in the conventionally pretreated group C. Both HF and silane alone had significant effects (p < 0.0001), but CAP alone did not (p = 0.9377). Significant interactions were found between silane and CAP (p = 0.0222), and HF and CAP (p = 0.0046). The combined effects exceeded individual effects. Although group C showed the highest BS, no significant interaction was found between HF and silane (p = 0.6270). Three-factor interactions were significant (p < 0.0001).

**Conclusion:**

In the setting used, CAP could not replace conventional pretreatment. The highest BS of a group without HF was achieved by combining silane with CAP. However, BS of this pretreatment was approximately 24% lower than that of the conventional pretreatment.

**Graphical Abstract:**

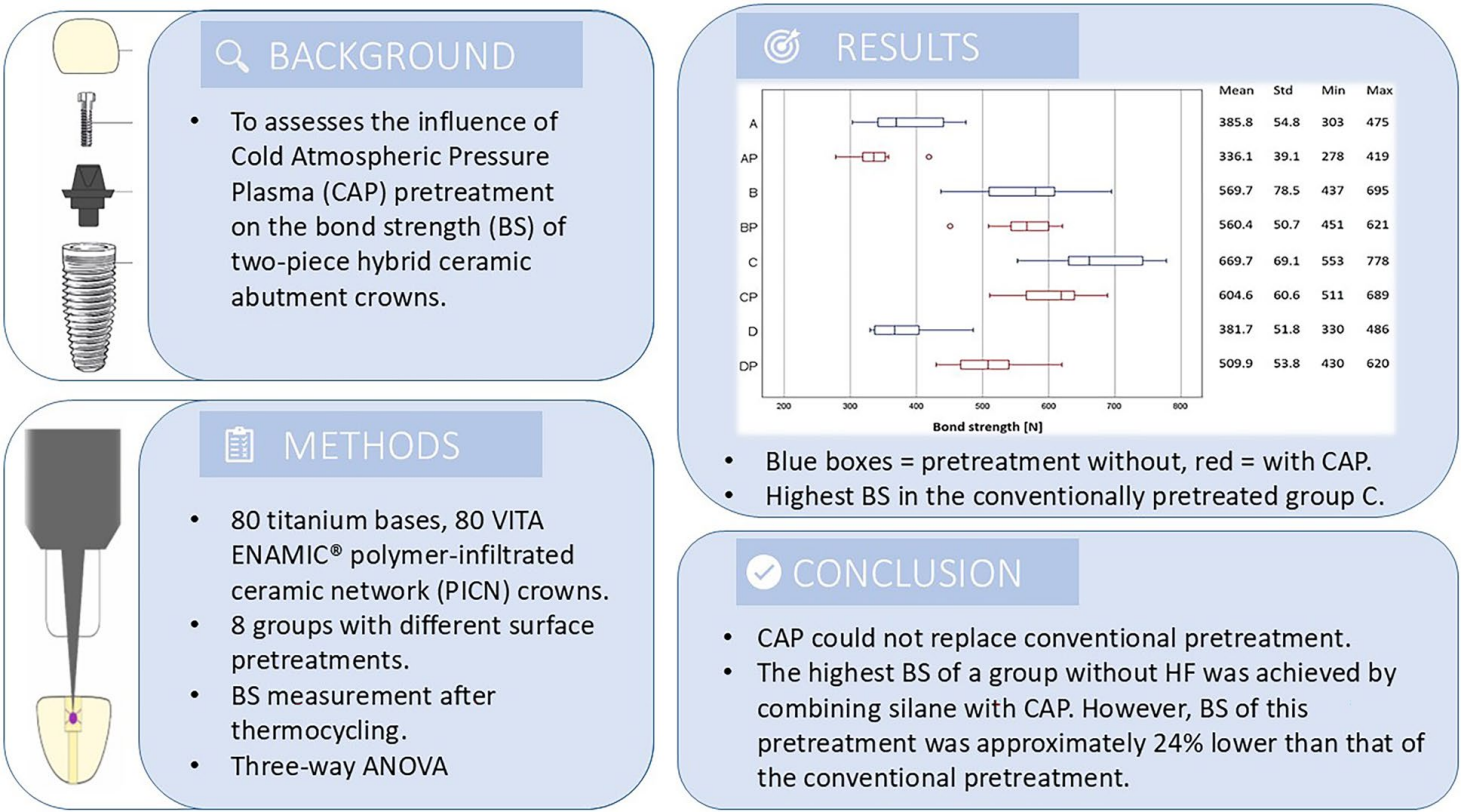

## Background

In implant prosthetics, two-piece hybrid abutment crowns play a significant role in replacing individual teeth. The CAD-CAM manufacturing of these crowns, which are bonded to prefabricated titanium bases (ti-bases), offers several advantages. These include access to nearly flawless industrially produced materials, improved precision and planning, better reproducibility, rapid and automatic data processing, and more efficient data storage [1]. Hybrid materials (also known as polymer-infiltrated ceramic networks PICN) for CAD-CAM systems have been developed, combining the aesthetic, durability and colour stability benefits of ceramics with the higher flexural strength and reparability of composite resins [2]. This combination offers high elasticity and the potential to absorb occlusal forces, which is beneficial for rigid implant restorations [3, 4]. Furthermore, these hybrid materials do not require a firing process, which simplifies the laboratory procedures. Most hybrid ceramics used for restorations are currently processed through subtractive milling [5].

Hybrid abutment crowns do not integrate the connection to the implant within the crown material itself. Instead, crowns are bonded to ti-bases [6]. The risk of adhesive failure has been reported, as only the ti-bases are directly connected to the implants with an abutment screw, while the link between the ti-bases and the crowns is based on the bonding protocol [7]. Therefore, the bonding surfaces of both components must be pretreated to ensure sufficient adhesive stability. For the titanium base, sandblasting with aluminium oxide particles (Al2O3) and applying an adhesive primer have proven effective [8]. However, excessive sandblasting can damage the ceramic structure of PICNs containing glass ceramics, and therefore, the use of HF etching followed by salinization has been recommended in those materials [9, 10]. In vitro studies conducted by the International Academy for Adhesive Dentistry (IAAD) have demonstrated that etching PICNs, such as VITA ENAMIC®, with 5% HF for 60 s and subsequently treating them with silane for an additional 60 s provides the optimal pretreatment protocol for the adhesive bonding of titanium bases to PICNs [11]. This pretreatment protocol is considered the “gold standard” for the adhesive bonding of titanium bases to PICNs. However, this method has been reported to be error-prone and difficult to handle: HF is a corrosive substance that can cause damage to the environment and human health [12]. It is therefore important to investigate alternative methods for the pretreatment of the PICNs surface.

CAP has gained attention in material science studies for its ability to enhance the wettability and bonding properties of various materials. CAP devices are designed for local pretreatment of a wide range of surfaces, including polymers, metals, ceramics, glass, hybrid materials and further applications [13].

The influence of plasma pretreatment prior to bonding crowns has been the subject of investigation in various studies [14–16]. However, these studies mainly involved zirconia crowns, treated with various methods, including plasma, and subjected them to water storage and thermocycling. Although plasma treatment increased the surface free energy, it did not change the surface roughness, leading to the conclusion that plasma treatment cannot fully replace sandblasting for zirconia surface treatment [17, 18]. A similar study by Görgen et al. on zirconia crowns involved plasma treatment of both the inner surfaces of zirconia crowns and the ti-bases before cementation [19]. In the present study, PICN crowns were subjected to plasma treatment because this area of research is still underexplored, and there are no standardised application guidelines for cold atmospheric plasma pretreatment of titanium bases and PICN crowns.

The objective of this study was to analyse the influence of CAP, both, alone and in combination with different other pretreatment methods, on the bond strength between the titanium bonding base and the PICN crown (VITA ENAMIC®). It is therefore also the objective to ascertain whether the conventional pretreatment can be complemented or partially replaced.

Two null hypotheses were formulated. The pretreatment measure has no influence on the BS between the CAD-CAM-fabricated PICN crowns and the ti-bases and the failure mode is not related to the surface treatment of the PICN crowns.

## Methods

### Production

**Fig. 1:**
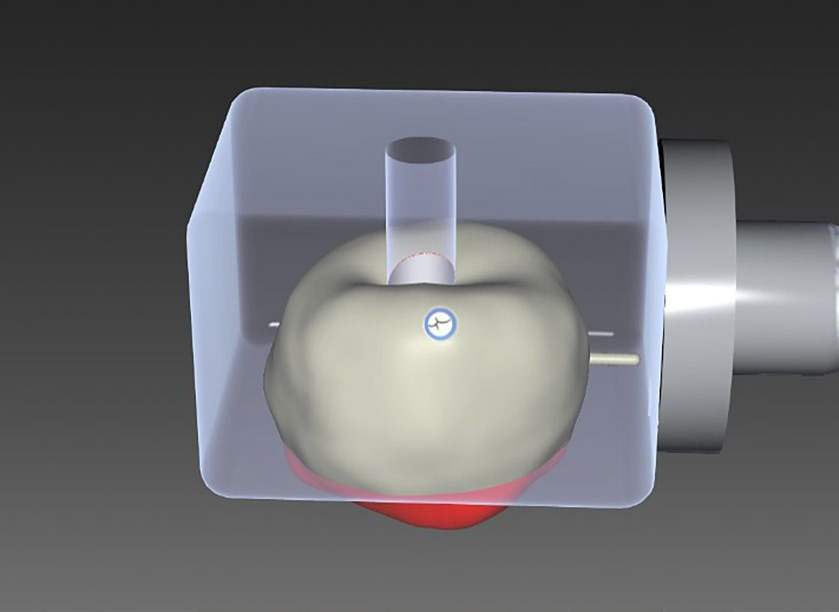
The design of the PICN crown incorporates design components that extend beyond the block (red marking)

In this study, a total of 80 ti-bases (Ti-Base NB RS 4.3 L, Sirona Dental Systems GmbH, Bensheim, Germany) and 80 Vita ENAMIC® PICN crowns (VITA ENAMIC^®^ for CEREC^®^/inLab, VITA Zahnfabrik, Bad Säckingen, Germany) were divided into 8 groups of 10 samples each. A similar test method was used in previous studies [19]. The materials for the bonding protocol and the conventional pretreatment protocol were used according to the manufacturer’s instructions from VITA Zahnfabrik. The crown was designed using CEREC SW® 5.0 software (Sirona Dental Systems GmbH, Bensheim, Germany). The milling blocks used consisted of a ceramic network (86 vol%) and a polymer network (14 vol%). The crown design was digitally positioned in the block so that part of the design was outside the block and the later milled crown had a flat basal structure (red marking) (Fig. 1). This design provided an even and flat support surface for the pull-off device (Zwick 1425, ZwickRoell GmbH & Co. KG, Ulm, Germany). The abutment in the PICN blocks was determined by the manufacturer, guaranteeing an identical shape and cement gap in all samples. To avoid contamination of the surfaces, gloves were worn during all procedures and changed between the different pretreatments.

In all groups, the ti-bases were pretreated in the same way as follows: Group (A)—(DP): sandblasting vertically to the ti-base at 10 mm distance using a dental sandblaster (P-G 400, Harnisch + Rieth GmbH & Co. KG, Winterbach, Germany) with 50 μm Al_2_O_3_ (Plurakorund, Pluradent AG & Co. KG, Offenbach, Germany), at 1.0 bar for approximately 10 s and application of the bonding agent (VITA ADIVA M-Prime, Metal/Alloy primer, Harvard Dental International GmbH, Hoppegarten, Germany) for 10 s. Different pretreatment protocols have been applied in the PICN crowns (Table 1).

**Table 1:** Pretreatment protocol and test groups

	Pretreatment of Ti-base (n = 80)	Pretreatment of PICN crown (n = 80)
Group A (n = 10)	Sandblasting + M-Prime	Disinfection with alcohol
Group B (n = 10)	Sandblasting + M-Prime	Hydrofluoric acid (HF)
Group C (n = 1 0)	Sandblasting + M-Prime	HF + silane (C-Prime)
Group D (n = 10)	Sandblasting + M-Prime	C-Prime
Group AP (n = 10)	Sandblasting + M-Prime	Plasma piezobrush PZ3 (CAP)
Group BP (n = 10)	Sandblasting + M-Prime	HF + CAP
Group CP (n = 10)	Sandblasting + M-Prime	HF + CAP + C-Prime
Group DP (n = 10)	Sandblasting + M-Prime	CAP + C-Prime

The PICN crowns in groups B, C, BP and CP underwent HF treatment (VITA Ceramics Etch 3 ml syringe, hydrofluoric acid 5% etch gel, VITA Zahnfabrik, Bad Säckingen, Germany). Specifically, the inner surfaces of the crowns in these groups were pretreated with HF for a duration of 60 s. At the end of the 60 s, the HF was completely removed with running water and then with a steam jet device (Triton SLA, Bremer Goldschlägerei Wilh. Herbst GmbH & Co. KG, Bremen, Germany). Afterward, the surfaces were dried with an oil-free, clean air flow. In group C, HF pretreatment was followed by conditioning with a silane bonding agent (VITA ADIVA C-Prime, Harvard Dental International GmbH, Hoppegarten, Germany) for 10 s. Overall, the inner crown surfaces of groups C, D, CP and DP were pretreated with the silane agent. First, a drop of the silane was placed in a sterile tray. The inner surface of the crowns was then evenly and thinly moistened with the bonding agent using an application brush. The silane was applied to the crown surface for 10 s. Afterward, the surface was gently treated with an oil-free, clean air flow until complete drying was achieved. In the groups AP, BP, CP and DP, plasma conditioning (CAP) (piezobrush PZ3, relyon Plasma GmbH, Regensburg, Germany) was performed with the piezobrush PZ3 plasma device (prior to silane application, Group CP, DP). The piezobrush PZ3 employs advanced piezoelectric direct discharge (PDD) technology to produce CAP [20]. The plasma unit was operated at 100% power (18 W, 240 V, < 50° C) for a period of 30 s throughout the pretreatment process. The needle nozzle for non-conductive materials was selected as the device for conditioning the inner surface of the PICN crowns to ensure uniform treatment of the inner surface of the crown.

### Adhesive cementation of hybrid abutment crowns

For adhesive bonding a dual curing composite resin was used (VITA ADIVA IA-CEM; Harvard Dental International GmbH, Hoppegarten, Germany). The abutments (TiBase NB RS 4.3, Sirona Dental Systems GmbH, Bensheim, Germany) were screwed into an implant analogue (NobelParallel Conical Connection RP 4.3 × 11.5 mm, Nobel Biocare Services AG, Zürich, Switzerland) and fixed in a plastic clamp (Mediplast AB, Malmö, Sweden). The components of the adhesive were mixed using automix cannulas (3 M Deutschland GmbH, Neuss, Germany), disposing of the first mixed portion for every single bonding procedure. The screw channel was sealed with cotton wool. The pretreated surfaces of the titanium adhesive abutment and of the PICN crown were then completely covered with a thin layer of adhesive cement. The PICN crown was carefully and accurately placed onto the ti-base until it was seated in the final position. Excess adhesive was removed from the screw channel with a microbrush. The bonded parts were clamped in a special hybrid abutment bonding aid (HPdent GmbH, Gottmadingen, Germany) to guarantee standardized pressure in all specimens. The crowns were then cured from all sides for 3–5 s at 1200 mW/cm^2^ with a light curing lamp (Elipar S10, 3 M Deutschland GmbH, Neuss, Germany). Excess cement was removed with a LeCron spatula (Henry Schein Dental GmbH, Langen, Germany). The bonded hybrid abutments were kept in the hybrid abutment bonding aid for 10 min. To avoid an oxygen inhibition layer, glycerine gel (Liquid Strip, Ivoclar Vivadent AG, Schaan, Liechtenstein) was applied to the adhesive gap, followed by light polymerisation for additional 30 s. The time was recorded with a stopwatch (GEFU GmbH, Eslohe, Germany). After 10 min, the bonded PICN crowns were removed from the fixation device and remained at room temperature for at least 24 h. Subsequently, the adhesive gap was polished with ceramic polishers operating at a maximum speed of 5000 rpm.

### Thermocycling

All bonded PICN crowns underwent thermocycling to simulate oral aging. The specimens were thermocycled using a device (Thermocykler Willytec, SD Mechatronik GmbH, Feldkirchen-Westerham, Germany) alternating between cold (+ 5.0 °C) and warm (+ 55.0 °C) distilled water. Each cycle consisted of 30-s exposures to these temperatures, followed by 5 s of dripping and transfer periods between water basins. This process was repeated for a total of 5000 cycles, each lasting 80 s. Following thermocycling, the specimens were stored in distilled water at a temperature of 23 °C until further processing.

### Pull-off bond strength test

**Fig. 2:**
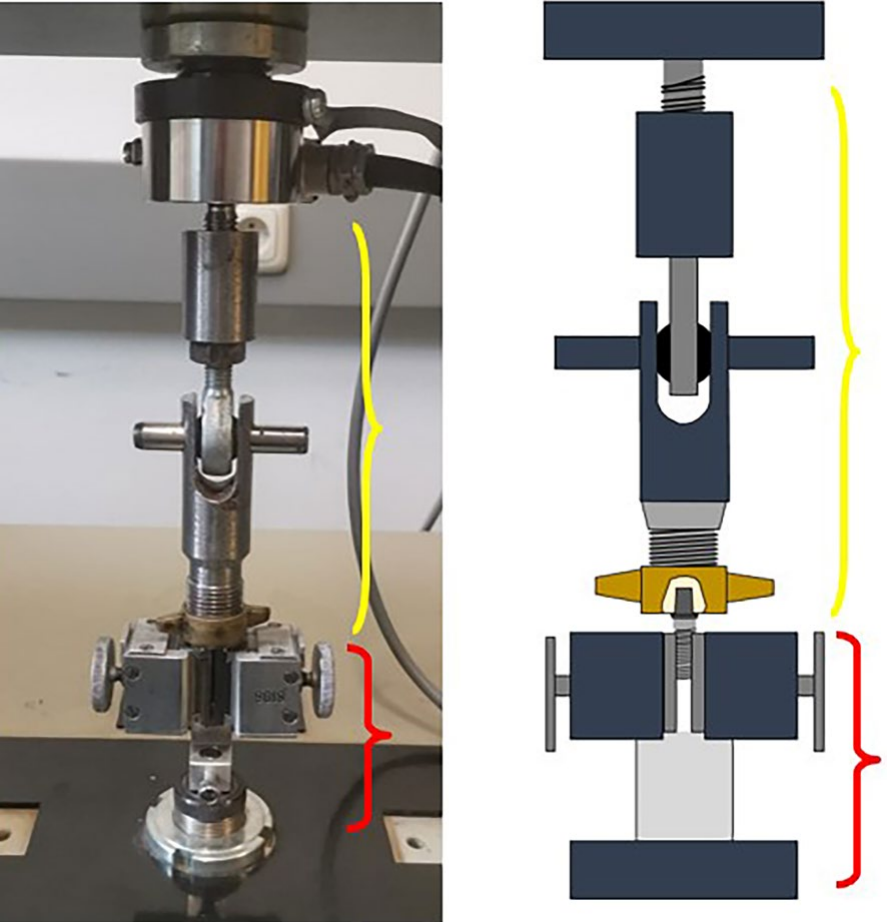
Custom-made holder (yellow) and wedge grip (red)

A universal testing machine (Zwick 1425, ZwickRoell GmbH & Co. KG, Ulm, Germany) was employed to perform pull-off tests with the objective of determining the maximum bond strength required to remove the crown. To prevent misinterpretation, the fracture cut-off threshold was set at 50 N. An implant (NobelParallel Conical Connection RP 4.3 × 11.5 mm, Nobel Biocare Services AG, Zürich, Switzerland) was secured using a wedge grip attached to the lower part of the machine, while the PICN crowns were placed in the upper part using a custom-made specimen holder designed specifically for this purpose (Fig. 2). The specimens were then screwed onto the implant with a torque of 35 Ncm. The holder ensured a flat contact surface by means of a polished steel disc with a central recess for the specimen. The gripping system uses a ball joint to ensure that the bond strength is applied straight and vertically, ensuring that the measuring axis corresponds to the test axis. The tests were conducted at a velocity of 1 mm/min, and were recorded on video for further analyses. All pull-off tests continued until the crowns fully detached from the adhesive bases.

### Detailed failure mode analysis

Following the completion of the pull-off tests, each surface of both components from the respective test groups underwent a detailed visual analysis using an optical microscope (VHX-1000, Keyence Deutschland GmbH, Neu-Isenburg, Germany) at 30× magnification, with images recorded for documentation. During the inspection, three observers identified any remaining adhesive residues and categorised fractures as either adhesive or cohesive. Adhesive fractures typically occur when the adhesive's maximum strength is exceeded. In cases of pure adhesive fracture, all adhesive remains on one substrate without any residue on the other. This can be observed in cases where the adhesive adheres entirely to the titanium bonding base or PICN crown. Furthermore, an adhesion fracture may occur on both components to be joined, whereby the adhesive residues on the two components to be joined fit together like a jigsaw puzzle.

### Statistical analysis

The statistical analysis of the data was carried out using the programmes IBM SPSS Statistics (SPSS Statistics Version 27, IBM Corp., Armonk, New York, USA) and SAS (SAS 9.4., SAS Visual Statistics, 100 SAS Campus Drive, Cary, NC, USA). With regard to the central questions the following null hypotheses were formulated and tested as part of a statistical analysis:

A three-factor ANOVA was performed to compare the mean value of the BS with regard to the various pretreatment measures and the interactions between the pretreatment components, in which the effect size was determined. A Shapiro–Wilk test and a Levene test were carried out beforehand to check whether the model assumptions of the ANOVA were significantly violated. The significance level was set at p = 0.05.

For the comparison of the mean BS based on the different failure modes, a Fisher’s Exact Test was carried out. The significance level was set at p = 0.05.

## Results

**Fig. 3:**
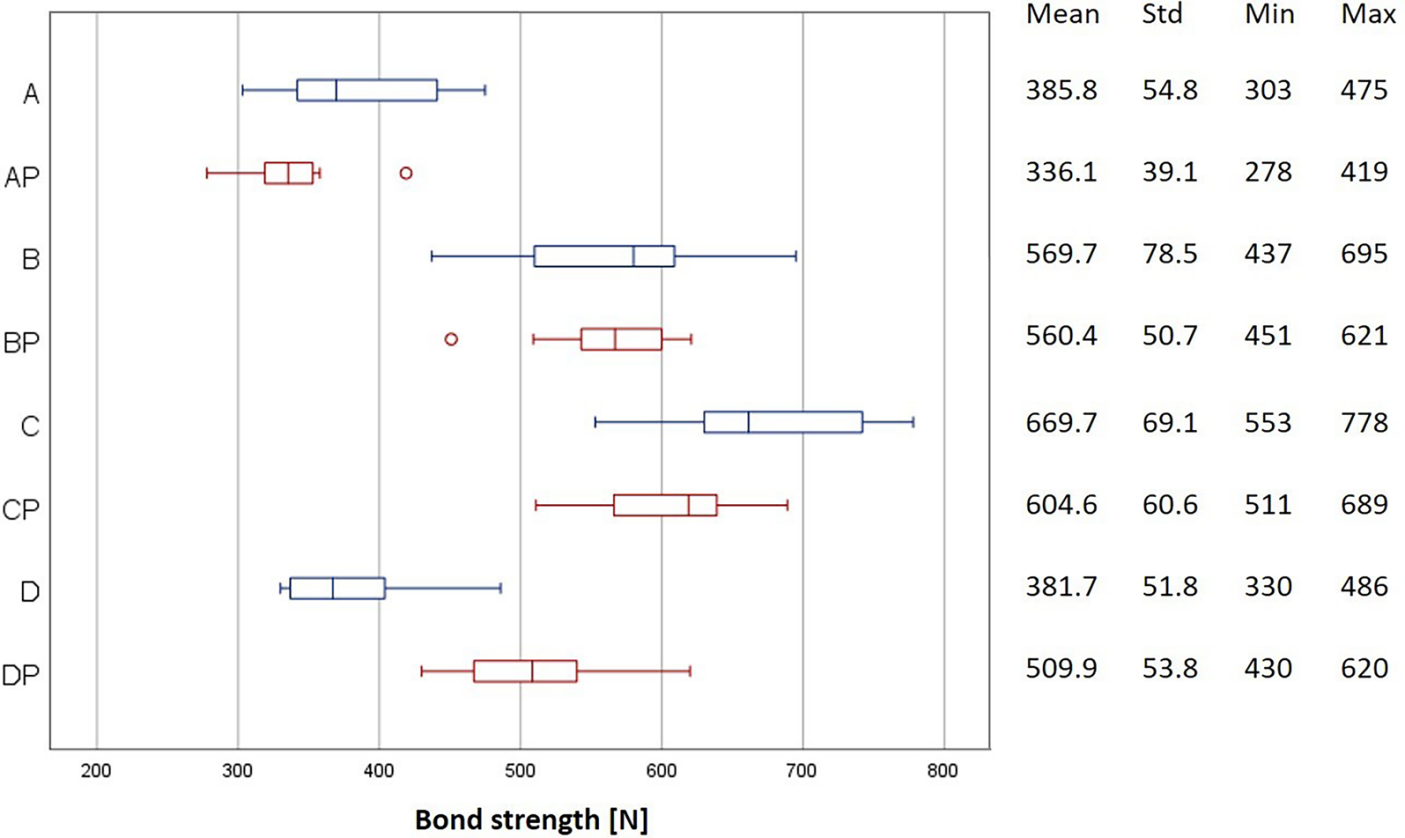
Box plot representation of the BS values in the respective groups, mean values, standard deviation (Std), minimum and maximum values

**Fig. 4:**
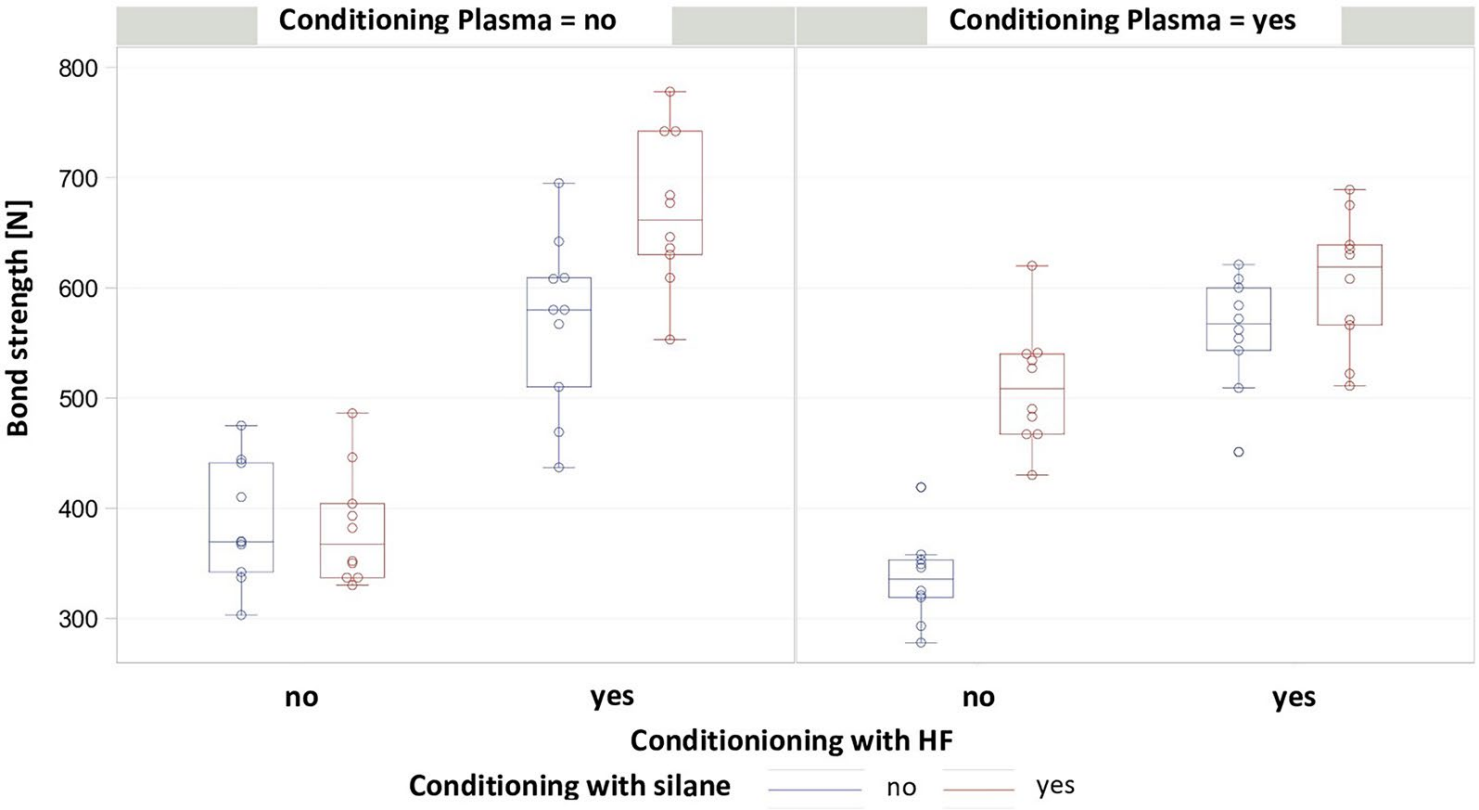
Comparison with versus without CAP with application of silane and HF

**Fig. 5:**
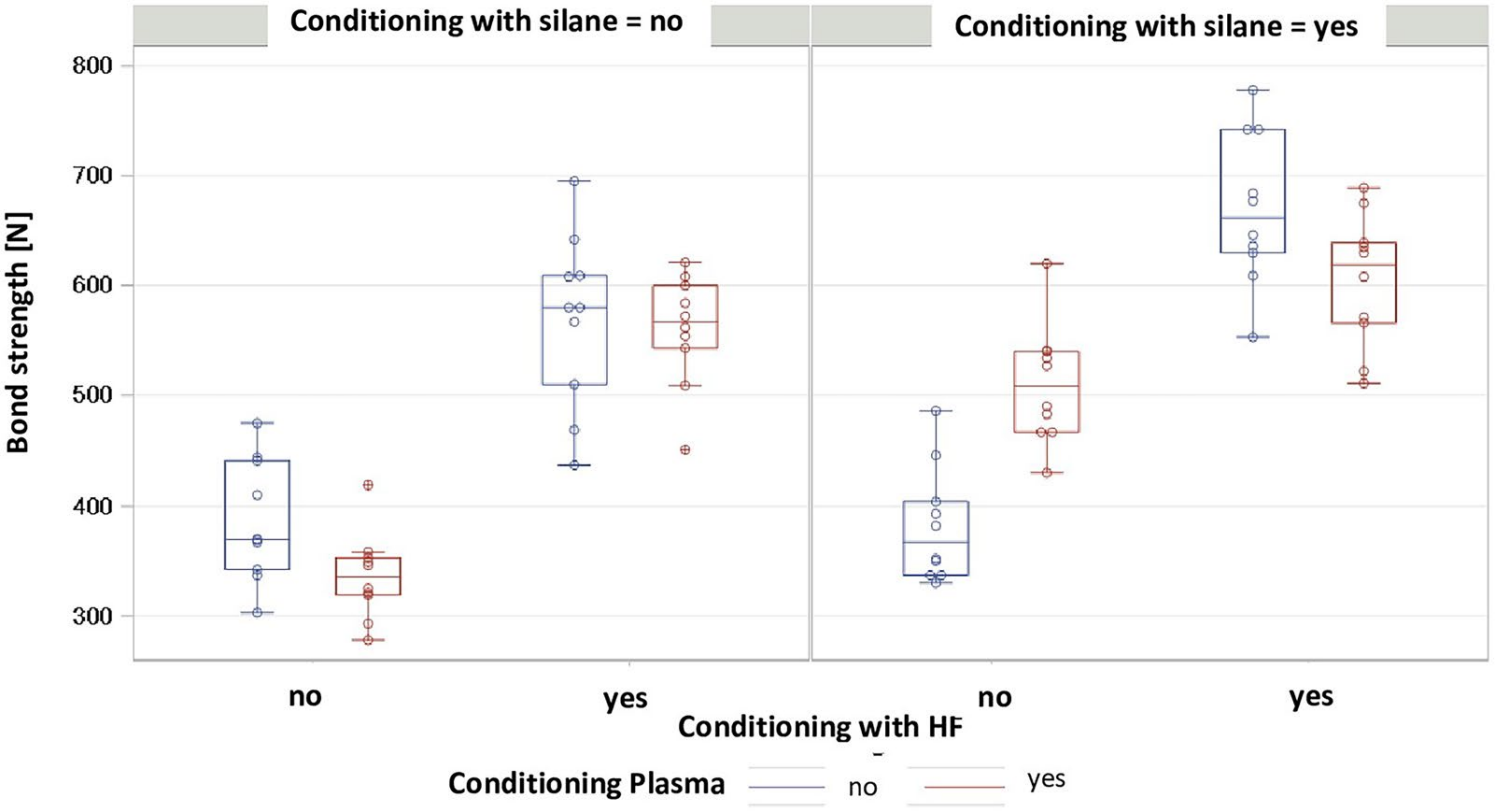
Comparison of groups with and without application of silane

The distribution of the BS values for each group is presented in Fig. 3. The mean pull-off forces ranged from 336.1 N ± 39.1 N in the AP group to 669.7 N ± 69.1 N in the C group. All groups showed normally distributed results (Shapiro–Wilk test, p > 0.05) and the variance between groups was homogeneous (Levene test, p = 0.3975). P-values and mean differences for pairwise comparisons between groups are shown in Table 2. The effects for silane (p < 0.0001) and of HF (p < 0.0001) were significant. CAP treatment had no significant effect on pull-of forces (p = 0.9377). CAP only had a significant effect in the comparison between group DP and group D, while it had no significant effect in the other groups. Significant interactions were observed between silane and CAP (p = 0.0222) and between HF and CAP (p = 0.0046). Three-way interactions were also significant (p < 0.0001). The application of 5% HF had a positive effect on BS (Fig. 4). Figure 5 shows the comparison of pull-off forces for the groups pretreated with and without silane, demonstrating that the pretreatment methods used in combination with silane showed higher BS values, although no significant interaction between HF and silane (the conventional pretreatment method) was observed (p = 0.6270).

**Table 2:** P-values and mean differences for pairwise comparisons between groups

p-value	A	B	C	D	AP	BP	CP	DP
A	–	183.9	283.9	− 4.1	− 49.7	174.6	218.8	124.1
B	< 0.0001	–	100	− 188	− 233.6	− 9.3	34.9	− 59.8
C	< 0.0001	0.0063	–	− 288	− 333.6	− 109.3	− 65.1	− 159.8
D	1.0000	< 0.0001	< 0.0001	–	− 45.6	178.7	222.9	128.2
AP	0.5539	< 0.0001	< 0.0001	0.6580	–	224.3	268.5	173.8
BP	< 0.0001	1.0000	0.0020	< 0.0001	< 0.0001	–	44.2	− 50.5
CP	< 0.0001	0.8820	0.2157	< 0.0001	< 0.0001	0.6924	–	− 94.7
DP	0.0003	0.3140	< 0.0001	0.0001	< 0.0001	0.5335	0.0119	–

The p-values are shown in the lower triangle of the matrix

The upper triangle contains the differences, calculated as the column mean minus the row mean [N]

### Failure mode analysis

**Fig. 6:**
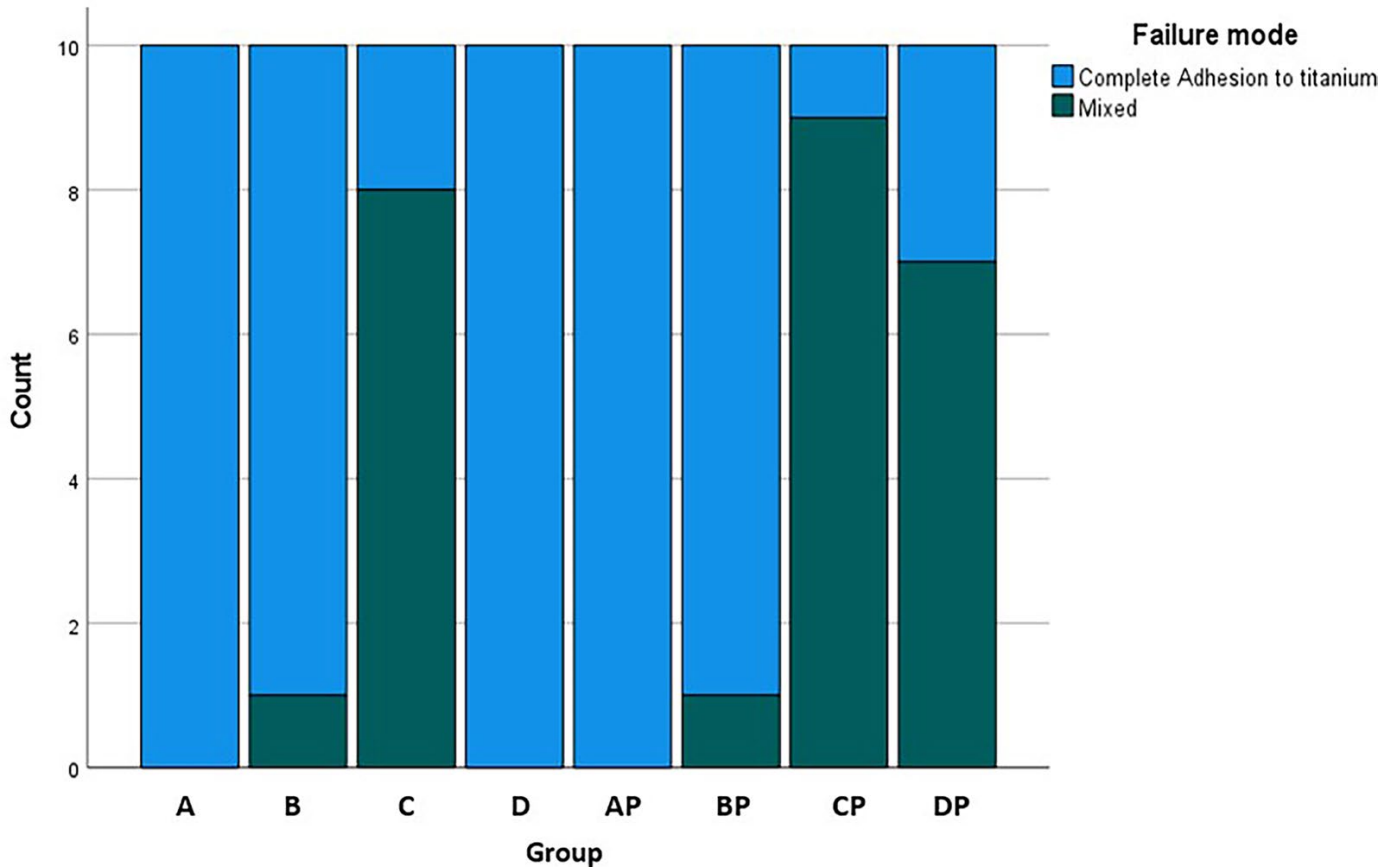
Graphical representation of the distribution of failure modes

**Fig. 7:**
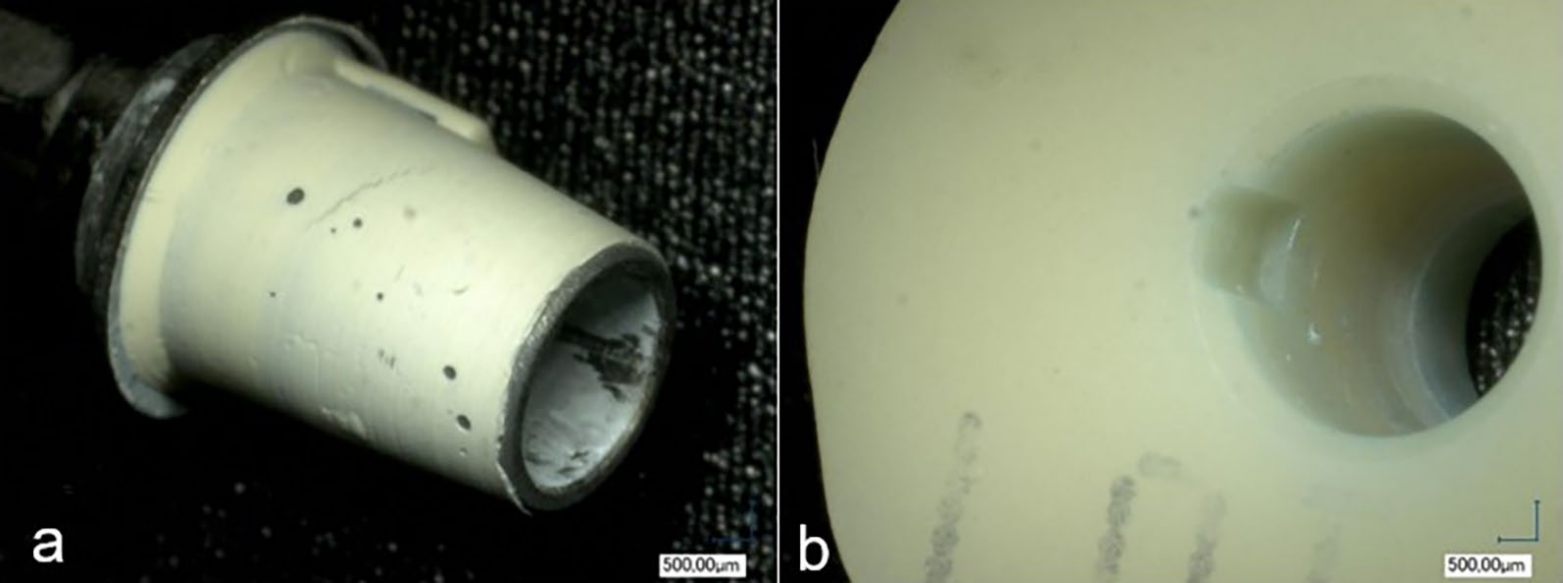
Adhesive fracture with adhesive residue completely on the titanium adhesive base. **a** Adhesive residue completely on the ti-base. **b** PICN crown surface free of adhesive residue

**Fig. 8:**
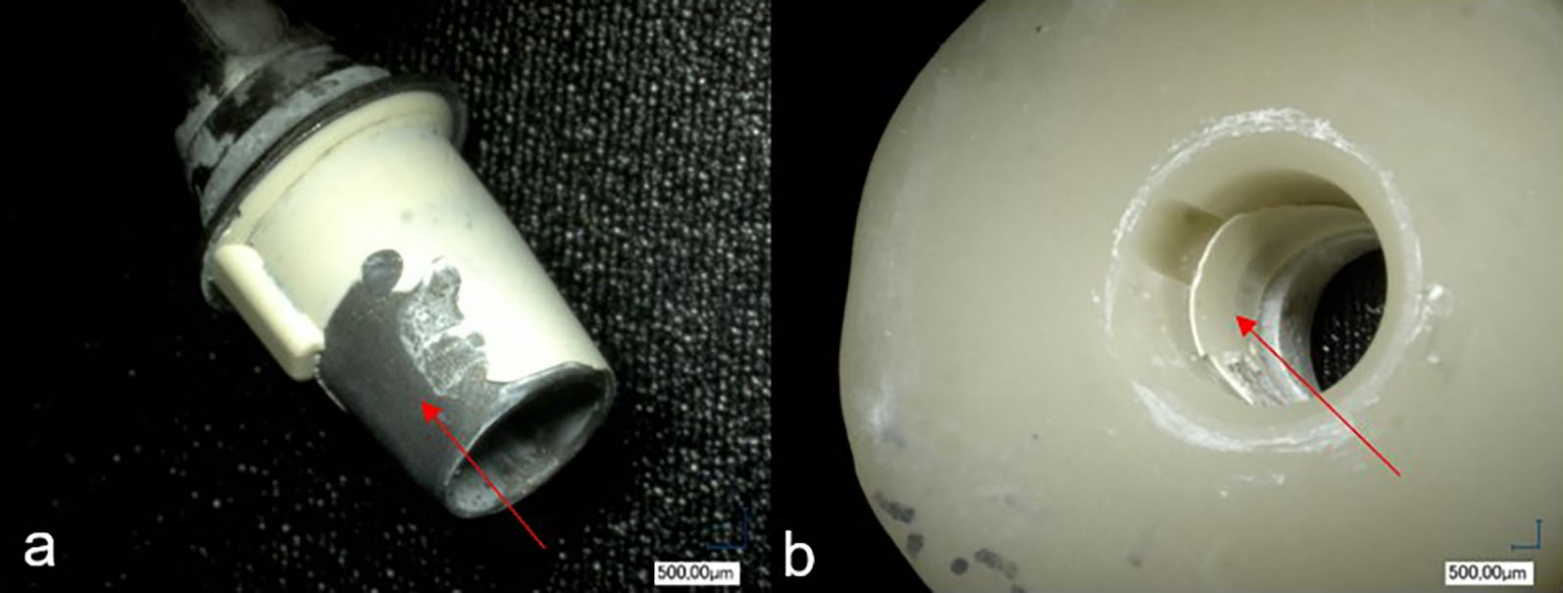
Adhesive fracture with adhesive residue on the ti-base (**a**) and on the PICN crown (**b**), puzzle-like (red arrow)

The different test groups produced different fracture patterns as shown in Fig. 6. There was a significant correlation between surface treatments and fracture patterns (p < 0.0001). In three groups the adhesive residue remained entirely on the ti-base, leaving the PICN crowns free of residue (Fig. 7). In group DP, 7 samples, in group C, 8 samples, and in group CP, 9 samples, had adhesive residues on both joining parts (Fig. 8). In two groups, only one specimen each had adhesive residue on the PICN.

## Discussion

In this study, Vita Enamic PICN crowns were subjected to a series of different pretreatments prior to bonding with ti-bases. All ti-bases were exposed to sandblasting and bonding agent treatment. The different pretreatment combinations for the PICN crowns including no pretreatment, HF, CAP treatment, and silane application. Following bonding, the samples were subjected to thermocycling, and subsequently underwent tensile shear tests to ascertain the BS. While silane and HF had a significant positive effect on bond strength (p < 0.0001 for both), CAP treatment alone did not (p = 0.9377). Three-way interactions were highly significant (p < 0.0001). The highest BS was measured in the conventionally pretreated group with HF and silane. Consequently, the two null hypotheses, which stated that pretreatment has no influence on BS and that the failure mode is not related to the surface pretreatment of the PICN crowns, were rejected.

A comparison of group B with control group A showed that mere HF pretreatment significantly improved the BS values. The four groups that achieved the four highest BSs were all groups in which HF (either alone or in combination with other agents) was also used. All groups pretreated with HF showed significant improvements (p < 0.0001). HF in combination with silane is the recommended gold standard for surface pretreatment of PICN crowns described in the literature [11]. HF is mainly recommended for etchable ceramics to improve surface roughness, wettability and micro retention while releasing hydroxyl groups that enable chemical bonding between the monomers. Prior studies have demonstrated that the application of 5% HF for approximately 60 s is an effective conditioning measure for improving the adhesion of the characterisation layer on the PICN [21–27]. The International Academy of Adhesive Dentistry (IAAD) recommends this procedure for PICN such as VITA ENAMIC^®^, followed by silane treatment [11]. The present study was able to confirm the effectiveness of the conventional pretreatment method for Group C (HF and silane), which also achieved the strongest bond. Interestingly, group D pretreated with silane alone did not show a significantly better bond strength compared to the control group A. The exclusive use of silane therefore had no influence. HF may have modified the surface of the PICN crowns, resulting in a better chemical bond when silane is subsequently applied, leading to a significant increase in bond strength.

In comparison of control group A, with group AP it can be seen that the use of CAP alone leads to a reduction in BS values, although this was not significant (p = 0.5539). Similarly, Görgen et al. observed comparable outcomes, noting that CAP treatment alone did not result in any improvement when bonding zirconia crowns [19]. In the present study, the BP and CP groups, in which plasma was used in addition HF (BP) and a combination of HF and silane (CP), CAP also did not show higher BS values compared to the groups without CAP treatment. The additional CAP treatment tended to result in lower values, but this was not significant.

Interestingly, when comparing group D, where the samples were pretreated with silane only, with group DP, where prior plasma treatment was added, it was found that in this combination CAP resulted in significantly higher BS values. In this combination, the effect of plasma and silane was modified by the interaction of the two individual factors as the combination of the effects was greater than the sum of the individual effects (p = 0.0222). The DP group achieved the fifth highest mean BS value of all groups and the highest mean value of the groups in which no HF was used. The results suggest that chemical bonding between the primer and adhesive surface, involving copolymerization with 3-MPS silane, forms Si–O-Si bonds through silanol groups. These bonds interact with the ceramic’s silicate portion, aided by the bonding agent's organic groups [28, 29]. This strong bond with the luting composite improves BS, as shown by higher BS values when the bonding agent is combined with HF, CAP, or both. These chemical interactions highlight the importance of proper pretreatment for the long-term stability of PICN crowns. This leads to the question of whether the pretreatment method of PICN with silane and CAP could replace the gold standard (HF and silane), as HF is harmful to the environment and health [12]. The utilisation of HF has been associated with a range of deleterious health outcomes, including dermal burns, eye damage, acute respiratory and gastrointestinal symptoms, and cardiac abnormalities during processing. These symptoms can occur if the substance comes into direct contact with the skin or eyes, is swallowed or inhaled [12]. A number of studies have demonstrated that CAP is unable to enhance the bond strength when used to bond zirconia crowns [14, 17, 18, 30, 31]. However, CAP has been observed to elevate the free surface energy of the samples examined [18]. The present study demonstrates that CAP does not enhance the bond strength when bonding PICN crowns with ti-bases. The combination of CAP and silane as surface conditioning of the PICN crowns results in an approximate 24% reduction in pull-off force compared to the gold standard, which contains HF. The acceptability of a significantly lower retention force to avoid HF would require verification through further studies, with the objective of determining whether there exists a specific threshold value of BS, beyond which the connection would remain permanently stable and to evaluate the possibility of whether this combination can indeed be a viable alternative to HF and provide a clinically adequate adhesive bond in the long term.

There were interesting differences in the distribution of failure modes between the groups. In groups A, D and AP, the adhesive residues remained completely on the titanium base in all samples after removal, while the interior of the PICN crowns was free of adhesive residues. These groups also had the lowest BS values. In the five groups in which adhesive residues also remained in the PICN crowns, significantly higher BS values were achieved, whereby a statistical correlation could be demonstrated. However, among the mixed fracture groups, there were two groups in which only one specimen each had adhesive residue on the crowns, and these two groups (B and BP) had higher BS values than other group DP in which seven crowns had adhesive residue. Although the two groups with the most mixed failure modes (C, CP) were the groups with the highest BS forces. That group B and BP also achieved high values, although 90% of their failure modes showed complete adhesion to the titanium base could be due to the influence of the HF used in these groups. It appears that above a certain BS threshold, the bond to the PICN becomes so strong that partially the bond to titanium becomes the weaker point, causing adhesive to remain on the ceramic. While the PICN bond is not the weakest link, increasing the bond strength to the PICN could further raise the average BS values. In the DP group, where silane and CAP conditioning were used, seven samples had adhesive on the crowns, but the average BS (510 N) was still lower than in groups B (570 N) and BP (560 N), suggesting that enhancing the PICN bond is key to improving overall bond strength. It also gives the impression that from a BS of 500–600 N, the bond to the PICN is so high that it is no longer solely responsible for the overall BS and not only the bond to the titanium is stronger.

In this study, the plasma piezobrush PZ3 was operated at full power (18 W) for 30 s throughout the entirety of the pretreatment process. The manufacturer recommended a working distance of 0.5–2 mm between the nozzle and the inner surface of the crown, which had an internal diameter of 3 mm. As demonstrated by Korzec et al., plasma pretreatment for 10 s at a distance of 1.5 mm was sufficient to activate the surface, with no further increase in activation area beyond this point [32]. Other studies have employed plasma for durations spanning from 15 to 80 s, with no evidence of thermal damage to the surface observed during these periods [19, 31, 33].

Pull-off tests were conducted using a Zwick 1425 universal testing machine, with the objective of measuring the maximum BS (Fmax) required to dislodge the crown or break the composite bond. This configuration, comparable to that employed in the study conducted by Görgen et al., involved fixing the specimens in a custom-designed holder, which is also used in this study [19]. The PICN crowns were mounted using a polished steel disc with a central recess, enabling flexible alignment to facilitate axis-oriented removal through ball joints. This design ensured linear and vertical force application, a crucial aspect as specimen alignment can influence pull-off behaviour, a factor previously observed [34].

The present study focused exclusively on VITA Enamic, a single type of PICN. Therefore, the results may not be applicable to other PICN materials with different compositions and properties. Furthermore, it is possible that other ceramics may respond differently to the surface pretreatments investigated. Additionally, only one plasma device (plasma piezobrush PZ3) operating at a specific power setting of 18 W was used. Plasma treatment outcomes can vary depending on the device, power setting, and exposure time. The use of a single device and fixed parameters restricts the understanding of how different plasma conditions might affect BS.

The thermal cycling protocol employed in this study adhered to established methods commonly used in similar research. [17, 19, 27, 28]. However, a notable limitation of the present study is that the samples were subjected solely to thermal aging, without incorporating mechanical aging processes, which could provide a more comprehensive evaluation of the material's long-term performance. A further limitation of this study is the absence of a sample size calculation, which may impact the statistical power and generalisability of the findings. The lack of an a priori determination of the appropriate sample size could affect the robustness of the results. Nevertheless, the applied sample size was sufficient to demonstrate statistically significant differences among the investigated pretreatment protocols.

Future studies should explore various plasma devices and settings including various PICN materials including printed materials for a more comprehensive understanding of its effects on bonding.

## Conclusion

Within the limitations of the study, it is demonstrated that the strongest BS was achieved with the conventional pretreatment with HF and silane, which therefore remains the recommended procedure. CAP treatment of the PICN crowns did not significantly increase BS, except in combination with mere silane, where a positive interaction could be seen. However, the BS with this pretreatment was approximately 24% lower than with the conventional pretreatment containing HF. Further investigation is necessary to clinically evaluate whether the BSs achieved without HF etching represent a valid alternative, avoiding HF etching in the pretreatment of PICNs.

## Data Availability

No datasets were generated or analysed during the current study.

## Abbreviations

3-MPS: 3-Methacryloyloxypropyltrimethoxysilane
Al2O3: Aluminium oxide
ANOVA: Analysis of variance
CAD: Computer-aided-design
CAM: Computer-aided-manufacturing
CAP: Cold atmospheric-pressure plasma
CEREC: : CERamic REConstruction
HF: Hydrofluoric acid
IAAD: International academy for adhesive dentistry
PICN: Polymer-infiltrated ceramic network
Si–O–Si: Siloxane
BS: Bond Strength
